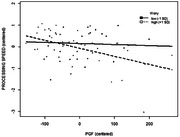# Worry differentially affects the impact of placental growth factor on processing speed in older adults with amnestic Mild Cognitive Impairment

**DOI:** 10.1002/alz.089876

**Published:** 2025-01-09

**Authors:** Jovian C Lam, Adriana Savettiere, Peter Louras, Jennifer Kaci Fairchild

**Affiliations:** ^1^ Stanford University School of Medicine, Stanford, CA USA; ^2^ Sierra Pacific MIRECC at VA Palo Alto, Palo Alto, CA USA; ^3^ Palo Alto University, Palo Alto, CA USA; ^4^ Veterans Affairs Palo Alto Health Care System, Palo Alto, CA USA

## Abstract

**Background:**

Placental growth factor (PIGF) is an angiogenic, pro‐inflammatory biomarker that is overexpressed in cardiovascular diseases. Recent literature has linked PIGF to the identification of cognitive impairment with white matter burden. Worry is associated with an increased risk for cardiovascular disease, accelerated aging and subsequent reduced brain volume, and decline in cognition. Given these associations, worry presents as a potential link to PIGF and cognitive impairments, though no research to date has examined its relationship to PIGF and cognition. Thus, current study aims to examine the possible moderating role of worry on PIGF and cognitive function (e.g., processing speed) in older adults with amnestic mild cognitive impairment (aMCI).

**Method:**

Participants included sixty‐six community‐dwelling older Veterans (M_age_ = 70.97 ± 8.66 years) who met criteria for aMCI. The sample was primarily male (95%), White (74%), and well‐educated (56% with bachelors or above). Composite scores reflective of processing speed were created using participants’ performances on the following measures: Symbol Digit Modalities Test, Stroop Color and Word subtests, and Trail Making Test A. Worry was measured by the Penn State Worry Questionnaire. Plasma PIGF levels were analyzed using SomaScan® disease‐specific panels. Hierarchical linear regressions were used to examine the moderating effect of worry on the relationship between PIGF and processing speed, while controlling for age.

**Result:**

Results indicated an overall significant model F(4,61) = 8.28, p < .01, ΔR^2^ = .03 and main effect of PIGF (β = ‐2.12e‐03, t = ‐2.28, p = .03) on processing speed. There was also a significant interaction between worry and PIGF (β = ‐1.30e‐04, t = ‐2.02, p < .05). Follow‐up analyses indicated a significant association between PIGF and processing speed in individuals with higher levels (+ 1 SD) of worry (β = ‐.01, t = ‐3.34, p < .01).

**Conclusion:**

The current results present a novel finding that worry intensifies the inverse relationship of PIGF and processing speed. Importantly, the current study reinforces a biopsychological link to cognition and a more nuanced consideration when examining biomarkers and risk factors of cognitive impairment.